# 西妥昔单抗联合化疗治疗晚期非小细胞肺癌的临床疗效分析

**DOI:** 10.3779/j.issn.1009-3419.2016.05.03

**Published:** 2016-05-20

**Authors:** 晟 杨, 燕 王, 兴胜 胡, 宏羽 王, 学志 郝, 建萍 许, 麟 汪, 彬 王, 峻岭 李, 龙妹 赵, 培娣 姜, 凤莲 屈, 湘茹 张, 远凯 石

**Affiliations:** 100021 北京，国家癌症中心/中国医学科学院北京协和医学院肿瘤医院内科，抗肿瘤分子靶向药物临床研究北京市重点实验室 Department of Medical Oncology, Beijing Key Laboratory of Clinical Study on Anticancer Molecular Targeted Drugs, National Cancer Center/Cancer Hospital, Chinese Academy of Medical Sciences and Peking Union Medical College, Beijing 100021, China

**Keywords:** 肺肿瘤, 西妥昔单抗, 靶向治疗, 化疗, Lung neoplasms, Cetuximab, Targeted therapy, Chemotherapy

## Abstract

**背景与目的:**

西妥昔单抗是作用于表皮生长因子受体的单克隆抗体。已有的研究表明在化疗基础上加用西妥昔单抗，可以提高晚期非小细胞肺癌患者的疗效，但来自东方人群的数据有限。本研究旨在分析西妥昔单抗联合化疗治疗中国晚期非小细胞肺癌患者的疗效。

**方法:**

纳入接受西妥昔单抗联合化疗的晚期非小细胞肺癌患者，回顾性分析其临床数据，包括患者的临床特征、疗效和不良反应。

**结果:**

共纳入40例患者，其中29例为男性，36例为腺癌，23例既往接受过姑息性化疗（中位2个化疗方案），上一个化疗方案的中位无进展生存期（progression-free survival, PFS）为2.3个月。采用西妥昔单抗联合化疗后，全组患者的缓解率为32.5%（13/40）。既往未接受过化疗和接受过化疗的患者缓解率分别为52.9%（9/17）和17.4%（4/23）（*P*=0.018）。全组患者的中位PFS为4.8个月。既往未接受过化疗的患者中位PFS为8.4个月，而接受过化疗者的PFS为4.1个月（*P*=0.062）。全组患者的中位总生存期为17.1个月。不良反应可处理，未发生治疗相关死亡。

**结论:**

西妥昔单抗联合化疗有望提高中国晚期非小细胞肺癌患者的疗效，并且不良反应可耐受。

## Abstract (en)

**Background and objective:**

Cetuximab is a monoclonal antibody directed against epidermal growth factor receptor. Emerging evidence showed improved efficacy with the addition of cetuximab to chemotherapy in advanced non-small cell lung cancer (NSCLC), but the data in oriental population are limited. The aim of this study is to investigate the efficacy of cetuximab in combination with chemotherapy in Chinese patients with advanced NSCLC.

**Methods:**

NSCLC patients receiving cetuximab in combination with chemotherapy in Cancer Hospital, Chinese Academy of Medical Sciences & Peking Union Medical College were enrolled and retrospectively analyzed. Clinical characteristic, efficacy, outcome and toxicity data were analyzed.

**Results:**

A total of 40 patients were enrolled into this study in which 29 were male, 36 with adenocarcinoma. In the 23 patients who had received palliative chemotherapy previously (with a median of 2 prior chemotherapy regimens), the median progression-free survival (PFS) after the last prior chemotherapy regimen was 2.3 months. For the overall population, 13 (32.5%) patients achieved partial response after cetuximab in combination with chemotherapy. Response rate were 52.9% (9/17) and 17.4% (4/23) in chemotherapy-naive patients and chemotherapy-treated patients, respectively (*P*=0.018). The median PFS was 4.8 months for the overall population. In chemotherapy-naive patients and chemotherapy-treated patients, the median PFS was 8.4 months and 4.1 months, respectively (*P*=0.062). The estimated median overall survival was 17.1 months. Toxicities were generally manageable and no treatment-related deaths occurred.

**Conclusion:**

Cetuximab in addition to chemotherapy appears to be associated with promising efficacy and acceptable toxicity profile in Chinese patients with advanced NSCLC. Further validation is needed.

晚期非小细胞肺癌（non-small cell lung cancer, NSCLC）是不可治愈的疾病。化疗是晚期NSCLC的重要治疗手段，但疗效已达平台期。分子靶向药物的出现显著提高了晚期NSCLC的疗效。表皮生长因子受体（epidermal growth factor receptor, EGFR）是晚期肺癌最有价值的治疗靶点之一^[1]^。针对EGFR的小分子酪氨酸激酶抑制剂（tyrosine kinase inhibitor, TKI）作为单药治疗晚期NSCLC时，具有较强的抗肿瘤效果^[2, 3]^；而在多项对照研究^[4-7]^中，其与化疗的联合使用并未明显提高化疗的疗效。

西妥昔单抗是嵌合型的单克隆抗体，可与肿瘤细胞表面的EGFR特异性结合，抑制其下游信号转导并介导抗肿瘤免疫反应^[8]^。近年来，国外开展了数项西妥昔单抗联合化疗治疗晚期NSCLC的临床研究^[9-12]^，结果表明，这一方法可以在一定程度上改善疗效。但此类方案治疗中国晚期NSCLC患者的数据仍十分有限。本研究分析中国医学科学院肿瘤医院采用西妥昔单抗联合化疗治疗NSCLC的数据，探讨其疗效。

## 1 资料与方法

### 1.1 患者一般资料

纳入2008年3月-2012年11月在中国医学科学院肿瘤医院接受西妥昔单抗联合化疗的NSCLC患者。所有患者经病理学或细胞学确诊为NSCLC，按美国癌症联合委员会（American Joint Committee on Cancer, AJCC）第7版分期标准判定为Ⅲb期或Ⅳ期，并有完整临床资料。

### 1.2 治疗方案

所有患者均接受西妥昔单抗联合化疗。西妥昔单抗首剂用量为400 mg/m^2^，静脉输注120 min；之后每周用药一次，每次250 mg/m^2^，静脉输注60 min。化疗方案的选择由临床医师根据患者病情个体化决定。

### 1.3 临床特征

通过查阅病历，获取患者的临床基线特征和治疗信息。本研究收集以下数据：患者性别、年龄、吸烟史、病理类型、开始治疗时的分期、行为状态、既往治疗史、联合西妥昔单抗的治疗方案、疗效和不良反应。

### 1.4 疗效评价

依照实体瘤疗效评价标准（Response Evaluation Criteria in Solid Tumors, RECIST）1.1版^[13]^，分为完全缓解（complete response, CR）、部分缓解（partial response, PR）、疾病稳定（stable disease, SD）和疾病进展（progressive disease, PD）。CR与PR须经第2次影像学检查确认。无进展生存期（progression-free survival, PFS）定义为自首次使用西妥昔单抗至首次记录的PD时间或死亡时间。总生存期（overall survival, OS）定义为自首次使用西妥昔单抗至患者死亡的时间。随访时间截至2016年4月7日。

### 1.5 安全性评价

按不良事件常用术语标准（Common Terminology Criteria for Adverse Events, CTCAE）4.0版评价不良反应。由于联合使用了不同化疗方案，重点评价与西妥昔单抗有关的不良反应。

### 1.6 统计学方法

采用SPSS 20.0统计学软件进行数据的统计学处理。计量资料采用中位数描述，计数资料采用率表示。率的比较采用卡方检验。生存数据采用*Kaplan*-*Meier*法并进行*Log*-*rank*检验。所有统计检验均为双侧概率检验，*P* < 0.05为差异有统计学意义。

## 2 结果

### 2.1 患者基线特征

本研究共纳入患者40例。其中男性29例（72.5%），女性11例（27.5%）；年龄中位50岁（33岁-72岁）；无吸烟史者26例（65.0%），有吸烟史者14例（35.0%）；腺癌患者36例（90.0%），鳞癌2例（5.0%），腺鳞癌1例（2.5%），另有1例（2.5%）为无法明确分类的NSCLC；原发病灶在左肺19例（47.5%），右肺21例（52.5%）；Ⅲb期6例（15.0%），Ⅳ期34例（85.0%）；行为状态评分90分者23例（57.5%），80分者12例（30.0%），70分者5例（12.5%）。

24例患者接受过针对晚期肺癌的全身性治疗（化疗或靶向治疗），16例接受西妥昔单抗联合化疗作为一线治疗。17例（42.5%）既往未接受过姑息性化疗，23例患者接受过姑息性化疗（既往接受过1个-4个化疗方案，中位2个化疗方案）；18例（45.0%）接受过EGFR-TKI（吉非替尼或厄洛替尼）治疗，其中11例接受过1种，7例接受过2种。13例（32.5%）接受过抗血管生成药物（贝伐珠单抗、重组人血管内皮抑制素或舒尼替尼）。11例（27.5%）接受过姑息性放疗。

对于24例接受过全身抗肿瘤治疗的患者，上一个治疗方案的缓解率为8.3%（2/24），中位PFS为1.5个月（0.7个月-2.3个月）。23例接受过化疗的患者中，上一个化疗方案治疗后3例达PR，缓解率为13.0%，中位PFS为2.3个月（1.7个月-2.8个月）。18例既往接受过EGFR-TKI患者中，上一次EGFR-TKI治疗的缓解率为5.6%（1/18），中位PFS为1.6个月（0.9个月-2.4个月）。

### 2.2 治疗方案

所有患者均接受了西妥昔单抗联合化疗。与西妥昔单抗联合的化疗方案包括含培美曲塞方案（19例，47.5%），含吉西他滨方案（9例，22.5%），含紫杉类方案（8例，20.0%）、含伊立替康方案（2例，5.0%）、含长春瑞滨方案（1例，2.5%）和含异环磷酰胺方案（1例，2.5%）。

### 2.3 缓解率

近期疗效见表 1。全组13例达PR，缓解率为32.5%。对于未接受过全身抗肿瘤治疗（包括化疗和分子靶向治疗）的患者，缓解率为56.2%，而接受过此类治疗者的缓解率为16.7%（*χ*^2^=6.857, *P*=0.009）；未接受过化疗患者的缓解率高于接受过化疗者，差异有统计学意义（分别为52.9%和17.4%，*χ*^2^=5.631，*P*=0.018）；接受过EGFR-TKI治疗的患者与未接受过EGFR-TKI者的缓解率分别为45.5%和16.7%（*χ*^2^=3.740, *P*=0.053）。

**1:** 患者接受西妥昔单抗联合化疗后的缓解情况 Response after cetuximab in combination with chemotherapy

	PR [*n* (%)]	SD [*n* (%)]	PD [*n* (%)]
Overall	13 (32.5)	17 (42.5)	10 (25.0)
Prior systemic therapy			
Yes	4 (16.7)	14 (58.3)	6 (25.0)
No	9 (56.2)	3 (18.8)	4 (25.0)
Prior chemotherapy			
Yes	4 (17.4)	14 (60.9)	5 (21.7)
No	9 (52.9)	3 (17.6)	5 (29.4)
Prior EGFR-TKI			
Yes	3 (16.7)	12 (66.7)	3 (16.7)
No	10 (45.5)	5 (22.7)	7 (31.8)
PR: partial response; SD: stable disease; PD: progressive disease; EGFR-TKI: epidermal growth factor receptor tyrosine kinase inhibitor.			

### 2.4 PFS

至末次随访时，38例患者已进展。全组患者的中位PFS为4.8个月（3.4个月-6.1个月）（图 1A）。在接受西妥昔单抗联合化疗作为一线治疗的16例患者中，中位PFS为8.4个月（5.2个月-11.7个月），而对于接受过化疗或靶向治疗的24例患者，其中位PFS为4.0个月（2.7个月-5.3个月），差异有统计学意义（*P*=0.039）。既往未化疗过患者的中位PFS与化疗过的患者相比，有更长的趋势：分别为8.4个月（4.1个月-12.8个月）和4.1个月（3.1个月-5.1个月，*P*=0.062）（图 1B）。既往未接受过EGFR-TKI治疗的患者，其中位PFS为6.0个月（0个月-12.5个月）；而接受过EGFR-TKI治疗者的中位PFS为4.4个月（3.0个月-5.8个月）。

**1:**
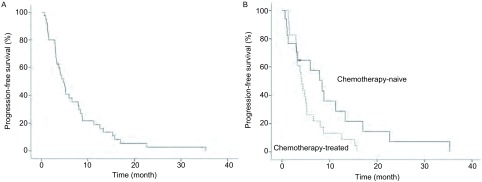
无进展生存曲线：全组患者（A）以及有化疗史和无化疗史的亚组（B） *Kaplan*-*Meier* estimates of progression-free survival for the entire population (A) and subgroups by history of chemotherapy (B)

### 2.5 OS

至末次随访时，35例患者已死亡。全组中位OS为17.1个月（13.3个月-21.0个月）（图 2A）。其中接受过化疗或靶向治疗患者的中位OS为14.3个月（10.3个月-18.2个月），未接受过化疗或靶向治疗的患者中位OS为26.1个月（17.1个月-35.2个月）。本研究前未接受过化疗与接受过化疗的患者，中位OS分别为26.1个月（17.9个月-34.4个月）和15.3个月（12.6个月-18.1个月）（图 2B）。对于未接受过EGFR-TKI和接受过EGFR-TKI治疗的患者，其中位OS分别为24.6个月（11.0个月-38.2个月）和15.3个月（11.8个月-18.9个月）。

**2:**
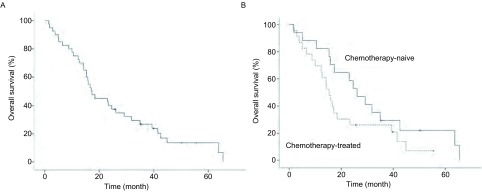
总生存曲线：全组患者（A）以及有化疗史和无化疗史的亚组（B） *Kaplan*-*Meier* estimates of overall survival for the entire population (A) and subgroups by history of chemotherapy (B)

### 2.6 不良反应

联合治疗方案的主要不良反应包括胃肠道反应、骨髓抑制等。所有不良反应经处理后恢复，无治疗相关死亡。与西妥昔单抗相关的不良反应为皮疹，包括1度、2度各9例（各22.5%）和3度5例（12.5%）。另有甲沟炎1例（2.5%），为1度。

## 3 讨论

本研究探讨了西妥昔单抗联合化疗治疗我国晚期NSCLC的疗效与安全性。由于西妥昔单抗在我国尚未获得晚期NSCLC的适应证，并且药品较为昂贵，此类方案在我国临床应用较少。本组是国内较大宗的报告，并且大部分患者已发生进展或死亡，PFS和OS数据较为成熟。结果初步表明，西妥昔单抗联合化疗治疗晚期NSCLC患者，可取得相对较高的缓解率、较长的PFS和OS。

在本研究中，对于未接受过全身性抗肿瘤治疗的患者，此方案的缓解率为56.2%，中位PFS与中位OS分别为8.4个月与26.1个月，均优于历史数据^[14]^。在FLEX（First-Line ErbituX in lung cancer）研究中，未接受过化疗的晚期NSCLC患者被随机分为两组，一组患者使用西妥昔单抗联合长春瑞滨和顺铂方案，另一组接受长春瑞滨和顺铂方案单纯化疗。结果显示，在化疗基础上加用西妥昔单抗能提高缓解率，延长至治疗失败时间（time to treatment failure）和OS^[11]^。但该临床试验选用的化疗方案较为陈旧，临床应用日益减少，且不良反应较为严重。而本研究提供了西妥昔单抗联合较新的化疗方案治疗晚期NSCLC的新数据，初步提示其疗效和安全性均较好。此外，在FLEX研究中，使用西妥昔单抗联合化疗后，东方人群的生存期明显较西方人群更长，而本研究中患者生存期同样较长，与之是相符的。

本研究中大部分患者接受过全身性抗肿瘤治疗。对于接受过化疗的患者，缓解率为17.4%，中位PFS为4.1个月。考虑到这些患者既往接受过中位2个化疗方案，本组疗效数据是相对理想的。鉴于本研究为单臂，我们采用同一组患者上一个化疗方案的缓解率和无进展生存期作为历史对照，进行探索性分析。对于既往接受过化疗的患者，西妥昔单抗联合化疗的缓解率与无进展生存期似乎均好于上一个化疗方案。临床研究^[15]^显示，西妥昔单抗联合伊立替康治疗对伊立替康耐药的晚期结直肠癌仍有一定疗效，提示西妥昔单抗具有逆转化疗耐药的作用。因此，值得探讨在NSCLC中，西妥昔单抗是否也具有逆转耐药的潜力。国外一项Ⅱ期临床研究^[16]^采用西妥昔单抗联合多西他赛治疗难治/耐药晚期NSCLC，缓解率为20%，中位至疾病进展时间（time to disease progression）为104 d。在Kim等^[17]^的Ⅲ期研究中，纳入了经含铂方案一线治疗后失败的晚期NSCLC患者，试验组或对照组作为二线治疗。试验组患者接受西妥昔单抗联合化疗（培美曲塞或多西他赛），对照组患者接受培美曲塞或多西他赛单药化疗，联合治疗组和单纯化疗组的缓解率、PFS和OS均无明显差异。遗憾的是，此研究中仅有8例亚裔患者接受了西妥昔单抗联合化疗，并且未报告这些患者的疗效数据。因此，其结论在中国患者中的适用性仍需要进一步证实。并且，Kim等^[17]^的研究也不能解答对于经二线以上化疗后的患者，化疗基础上加用西妥昔单抗能否提高疗效。

本研究表明，西妥昔单抗联合化疗治疗晚期NSCLC的安全性较好，但西妥昔单抗会在一定程度上加重不良反应。寻找合适预测指标来预判疗效和不良反应，提高治疗的获益/风险比，仍然是此类方案需要解决的重要问题。

本项研究的不足之处在于为回顾性，可能存在偏倚。并且，样本量仍需要进一步积累，以得到更稳健的结果。

总之，西妥昔单抗联合化疗有望提高中国晚期NSCLC患者的疗效，并且不良反应可耐受。这一结论需要更大样本的前瞻性研究证实。
